# CRMnet: A deep learning model for predicting gene expression from large regulatory sequence datasets

**DOI:** 10.3389/fdata.2023.1113402

**Published:** 2023-03-14

**Authors:** Ke Ding, Gunjan Dixit, Brian J. Parker, Jiayu Wen

**Affiliations:** ^1^Division of Genome Science and Cancer, John Curtin School of Medical Research, Australian National University, Canberra, ACT, Australia; ^2^School of Computing and Biological Data Science Institute, Australian National University, Canberra, ACT, Australia

**Keywords:** deep learning, big data, gene expression, yeast, genomics, HPC

## Abstract

Recent large datasets measuring the gene expression of millions of possible gene promoter sequences provide a resource to design and train optimized deep neural network architectures to predict expression from sequences. High predictive performance due to the modeling of dependencies within and between regulatory sequences is an enabler for biological discoveries in gene regulation through model interpretation techniques. To understand the regulatory code that delineates gene expression, we have designed a novel deep-learning model (CRMnet) to predict gene expression in *Saccharomyces cerevisiae*. Our model outperforms the current benchmark models and achieves a Pearson correlation coefficient of 0.971 and a mean squared error of 3.200. Interpretation of informative genomic regions determined from model saliency maps, and overlapping the saliency maps with known yeast motifs, supports that our model can successfully locate the binding sites of transcription factors that actively modulate gene expression. We compare our model's training times on a large compute cluster with GPUs and Google TPUs to indicate practical training times on similar datasets.

## 1. Introduction

Cis-regulatory sequences, also referred to as cis-regulatory modules (CRMs), are composed of promoters, enhancers, silencers, and insulators (Davidson and Erwin, 2006). These sequences are recognized and bound by DNA-binding regulatory proteins, called transcription factors (TFs), to control gene expression (Ni and Su, 2021). Alterations to the cis-regulatory sequences can affect their interaction with transcription factors, thereby influencing cell phenotype and cell-state transitions (de Boer et al., 2020). Increasing evidence demonstrates the significance of cis-regulatory element modification in relation to numerous diseases (Mathelier et al., 2015). Thus, understanding how cis-regulatory elements regulate gene expression has become crucial for understanding transcriptional gene regulation.

However, it has been extremely difficult to directly predict expression from DNA sequences due to a lack of training data that covers the entire potential sequence space. Recently, more than 100 million random promoter sequences and their corresponding expression levels have been identified in a high-throughput manner by measuring the expression output of the sequences regulating yeast gene constructs using Gigantic Parallel Reporter Assay (GPRA) (de Boer et al., 2020). This dataset provides the large training sets necessary for developing models to help decode the cis-regulatory logic. These random sequences, by chance, included multiple functional transcription factor binding sites (TFBS). By fitting an interpretable predictive (linear) model, the determinants of TF binding that predict gene expression could be studied. The use of random promoter sequences allowed an unbiased study of these determinants as they uniformly covered the entire potential sequence space, as opposed to real (native) promoter sequences that have evolved to favor a subset of sequences. In a follow-up study, Vaishnav et al. (2022) used this same dataset, using both random and native sequences, to study global hypotheses on patterns of TFBS evolution. For this study they used a more predictive transformer deep neural network (DNN) model to detect more complex gene regulatory patterns.

Several other deep neural network models have been developed to analyze the functional effects of DNA sequences. One such model is DeepSEA, which uses a convolutional neural network and was trained on genomic sequences and large-scale chromatin-profiling data. It has the ability to learn the regulatory sequence code related to chromatin effects (Zhou and Troyanskaya, 2015). Another model, DanQ, is a hybrid deep learning model that combines a convolutional neural network and a bidirectional long short-term memory recurrent neural network to identify the functional effects of DNA sequences (Quang and Xie, 2016). DeepATT builds on the DanQ model by incorporating a category-attention layer that focuses on feature representation for various DNA functions (Li et al., 2021). Basenji, which uses a dilated convolutional layer to identify long-range relationships, can predict sequential regulatory activity using genomic sequences over 100 kb as input (Kelley, 2020). SATORI is a deep learning model primarily utilizing self-attention layers to detect interactions between regulatory elements, such as transcription factor binding motifs, within genomic sequences (Ullah and Ben-Hur, 2021). Finally, Enformer, which uses a convolutional neural network to capture sequence motifs and a transformer network to model long-range interactions, can effectively predict gene expression based on genomic sequences (Avsec et al., 2021).

Concurrently with the research and development of increasingly deep and complex DNN architectures, the development of parallel acceleration hardware such as graphical processing units (GPUs) and tensor processing units (TPUs), and high performance clusters/cloud computing, enables the training of these more complex models by reducing the overall training time (Wang et al., 2020). As neural networks are becoming more sophisticated and the volume of scientific data keeps growing, the model training time on these different high performance computing (HPC) architectures is an important issue.

In this study, we propose a novel DNN model (CRMnet) for predicting the expression levels of yeast promoter DNA sequences, which achieves a Pearson correlation coefficient of 0.971 in the test dataset, improving upon the benchmark models proposed in Vaishnav et al. (2022). By accurately predicting the expression from promoter sequences, such models can be used predictively to design new regulatory sequences in synthetic biology, study the predicted effects of mutations (Vaishnav et al., 2022), and, by interpreting the model, help in understanding the determinants of gene regulation. Here, we interpret the model by visualizing saliency maps, showing we are able to identify key regions in the promoter sequences which most affect the corresponding expression. We demonstrate that our model can learn biologically meaningful information by quantifying the saliency information over known yeast sequence motifs. We compare the performance of our model on large datasets on parallel hardware of graphical processing units (GPUs) and tensor processing units (TPUs) on a HPC cluster.

## 2. CRMnet: Sequence-to-expression deep learning model

In a recent study (Vaishnav et al., 2022), the gene expression driven by millions of synthetic, randomly generated promoter sequences was experimentally determined. The synthetic promoter sequences, which were 80 base pairs in length and comprised of randomly sampled A, C, G, and T bases, were embedded in a promoter construct and their resulting expression was assayed in yeast (*Saccharomyces cerevisiae*) through high-throughput sequencing. The use of synthetic promoters greatly expanded the range of possible regulatory sequences, providing a diverse and representative dataset of transcriptional regulation for analysis.

In this study, we present a new deep learning model called CRMnet, which is designed to predict the expression level of yeast promoter DNA sequences with improved accuracy compared to previous models (Vaishnav et al., 2022), using the dataset of millions of synthetic promoter sequences. The model is based on a transformer-encoded U-Net architecture. The U-Net architecture consists of an initial encoding stage which extracts feature maps at progressively lower dimensions, optimized for the detection of transcription factor binding sites (TFBS) within the promoter sequence. A matching decoder stage upscales these feature maps back to the original sequence dimension, whilst concatenating with the higher resolution feature maps of the encoder at each level to retain prior information despite the sparse upscaling. This approach decodes a feature map at base-level precision.

The CRMnet model additionally includes a transformer encoder stage after the convolutional encoder that employs the self-attention mechanism to extract more global dependency information (Dosovitskiy et al., 2020) than convolutional layers, which is a key advantage over U-Net. The U-Net model (Ronneberger et al., 2015) is a fully convolutional neural network that primarily uses the convolution operation to learn the information embedded in genomic sequences, but has a limited receptive field and is less effective for learning global information and long-range dependencies such as interactions between TFBS.

**Figure 1:**
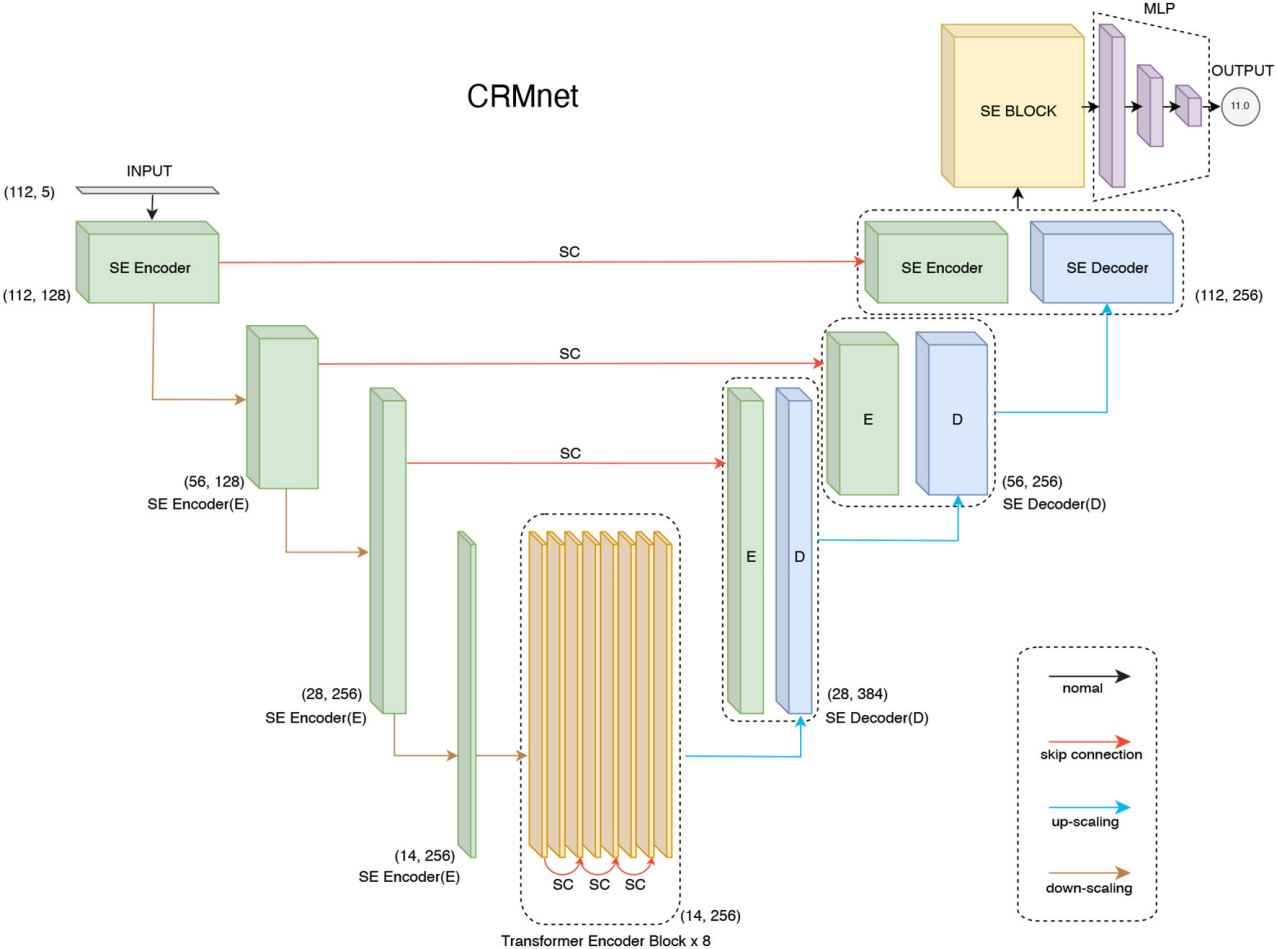
CRMnet model's architecture. Our CRMnet consists of Squeeze and Excitation (SE) Encoder Blocks, Transformer Encoder Blocks, SE Decoder Blocks, SE Block and Multi-Layer Perceptron (MLP). Similar to the U-Net architecture, the encoder and corresponding decoder at the same level have a skip connection (SC) so the decoder utilizes the concatenation of the upsampled feature map with the corresponding higher resolution encoder feature map at that level.

The CRMnet model consists of four key components (Figure 1): 1D convolutional neural network-based encoders to extract neighboring features in the input DNA sequences, a transformer encoder to extract longer range dependencies in the input sequence, 1D convolutional neural network-based decoders, with skip connections, to project the extracted features to the original sequence input dimension, and a multi-layer perceptron to predict the expression levels from the extracted features. In total, our model contains 34,170,289 parameters, of which 34,165,681 parameters are trainable and 4,608 parameters are not trainable (batch normalization layers, which only update the mean and variance, were not changed during backpropagation), and no parameters were frozen during transfer learning.

### 2.1. 1D CNN-based encoder

Our CNN-based encoder is built to extract TFBS motifs from genomic data inputs. The input is one-dimensional length 112 bp DNA sequences (80 bp promoter sequence plus padding, see Section 4) which is one-hot encoded (A,C,G,T + N for padding). 1D convolutional layers then learn filter parameters to extract predictive features from combinations of adjacent bases, with a filter size set to 11 in order to cover the average length of TFBS motifs (Stewart et al., 2012). Additionally, we add a squeeze excitation layer after each 1D convolutional layer because the squeeze and excitation operation has been demonstrated to improve the overall performance of CNN-based models by assigning importance scores to the different feature maps (Hu et al., 2018). Moreover, the original U-Net encoder block (Ronneberger et al., 2015) is modified by performing the down-sampling with a stride two convolution operation instead of max pooling, as the additional model parameterization has been shown to improve performance (Springenberg et al., 2014).

### 2.2. Transformer encoder

The transformer encoder accepts the CNN-based encoder's down-scaled feature maps as input. Each individual transformer encoder block follows the vanilla transformer architecture which is made up of a position-wise feed-forward network and a multi-head self-attention feed-forward network (FFN) module (Vaswani et al., 2017). Within each module, residual/skip connections and layer normalization are utilized in order to train a deeper neural network.

Unlike convolutional neural network stages which implicitly extract dependency information in local neighborhoods through the use of fixed-size kernels, transformer encoders use self-attention to extract global dependency information across the inputs, while explicitly encoding the positional information embedded into the input. Similar to other transformer-encoded models, we embed the positional information of the down-scaled input vectors (length 14) to our transformer encoder (Chen et al., 2021). We represent the positional information using sinusoidal position encodings and add it to the input token before feeding it to the transformer encoder.

### 2.3. 1D CNN-based decoder

The CNN-based decoder blocks are very similar to the original U-Net decoders, which use up-sampling to learn the representation (Ronneberger et al., 2015). Using a 1D transpose convolution operation to up-sample the resolution and attaching a squeeze excitation layer after each convolutional layer to up-weight the critical feature maps are the main differences in our implementation. The outputs are then concatenated with the skip connections from corresponding encoder levels to compensate for the potential loss of spatial information during downsampling.

### 2.4. SE block

It has been demonstrated that using Squeeze-and-Excitation (SE) blocks can significantly improve the generalization power of CNN-based models and achieve significant performance enhancements in several state-of-the-art CNN models with negligible increase in computational cost (Hu et al., 2018). The SE Block will initially compress the input feature map generated by learned convolutional filters using global average pooling. The channel-specific statistics will then be forwarded to the excitation operation, which will utilize two non-linear, fully connected layers to highlight the key channels. In other words, the SE Block can be regarded as a channel-specific self-attention function that compensates for the inability of the convolution operator to model the relationship among channels. As a result, we decided to adopt the SE operation after each convolutional layer and added a SE block in order to empower our model to focus on channel-specific feature responses of the convolution layers.

### 2.5. MLP

Our model will learn the expression levels from the extracted features utilizing a multi-layer perceptron (MLP). The fully connected dense layer will learn the non-linear combination of the extracted features from preceding layers. In the hidden layer of MLP, we use ReLU as the activation function (where alpha equals 0.1). Then, a linear activation is used to make the prediction of the expression levels in the final output neurons. To avoid overfitting, each dense layer is followed by a dropout layer. The first two dropout layers' dropout values are equal to 0.2 and the rest have dropout values equal to 0.1.

### 2.6. Pre-training and fine-tuning of models

We utilized a transfer learning approach to improve the performance of CRMnet. Specifically, to utilize the largest possible training set we pre-trained a more general model on a large dataset of randomly sampled data combining datasets from yeast grown in two different media types (“complex” and “defined” from Vaishnav et al., 2022). The pre-trained dataset contains over 50 million promoter sequences and their corresponding expressions. We then conducted a fine-tuning training stage in which the pre-trained model is retrained on the complex medium samples only, as used in the test sets of our study. During this stage, the pre-trained model's parameters were all unfrozen and trainable. The pre-trained model weights serve as good initializations for the fine-tuning on specific datasets, which can improve the model's performance on the target task (You et al., 2021). This method of transfer learning, though requiring an extra step and taking longer to train, can further improve the model's performance as demonstrated in **Figure 4**.

## 3. Results and discussion

We first evaluate the predictive performance of our model and compare the performance to that of existing deep learning models. We then use ablation studies to understand the roles of the subparts of our model. To demonstrate the biological significance of our model, we further apply saliency maps for model interpretation and compare with enriched transcription factor binding site motifs discovered by probabilistic motif discovery. Finally, we compare the training time between TPUs and GPUs.

### 3.1. Performance evaluation

**Figure 2:**
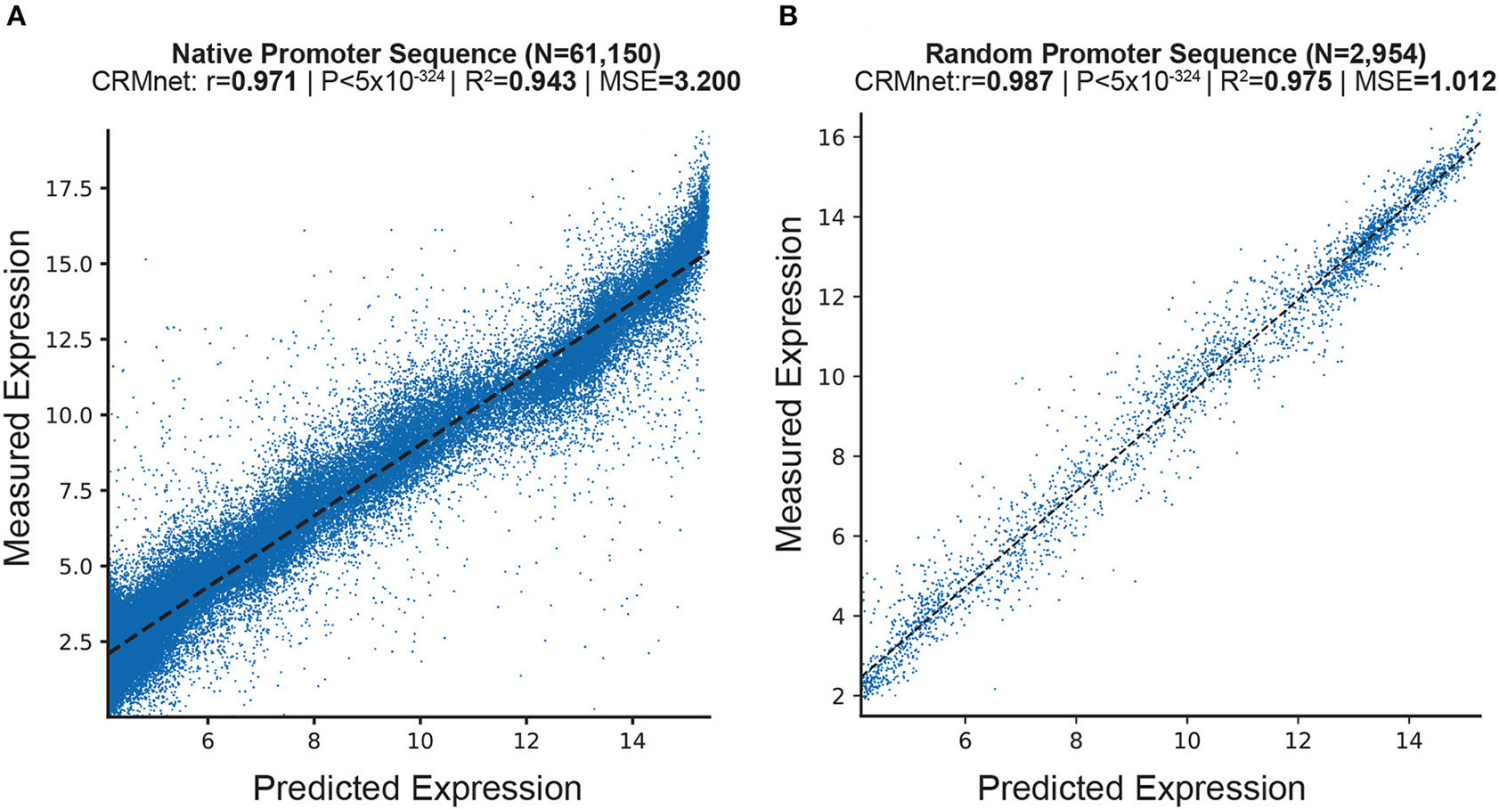
Prediction of expression from yeast native sequences from CRMnet. CRMnet tested on **(A)** native promoter sequences; and **(B)** random promoter sequences. The y-axes represent measured expression levels, while the x-axes represent predicted expression levels. As a benchmark, the model performance metrics of the Pearson *r*-value, associated two-tailed *p*-values, R-square, and MSE for the transformer model from Vaishnav et al. (2022) showed: **(A)**: *r* = 0.963, *P* < 5 × 10^−324^, *R*^2^ = 0.927, MSE = 3.965; **(B)**: *r* = 0.978, *P* < 5 × 10^−324^, *R*^2^ = 0.95, MSE = 1.425.

We here first present the predictive performance of our fine-tuned deep learning model on independent experimental test sets of both random and native (i.e., wild-type sequences found in yeast) promoter sequences, in both complex and defined mediums datasets (see Section 4). To evaluate the performance of the deep learning models on the test datasets, we measured the Pearson Correlation Coefficient (*r*), Coefficient of Determination (*R*^2^), Mean Squared Error (MSE), and Mean Absolute Error (MAE). The results show that our fine-tuned CRMnet model achieved excellent prediction performance on both native and random sequences (*r* = 0.971, and *r* = 0.987; MSE = 3.2; and MSE = 1.01, respectively) in complex medium (Figure 2 and Supplementary Tables 1, 2) and in defined medium (*r* = 0.955 and *r* = 0.973, respectively, Supplementary Figure 1).

**Figure 3:**
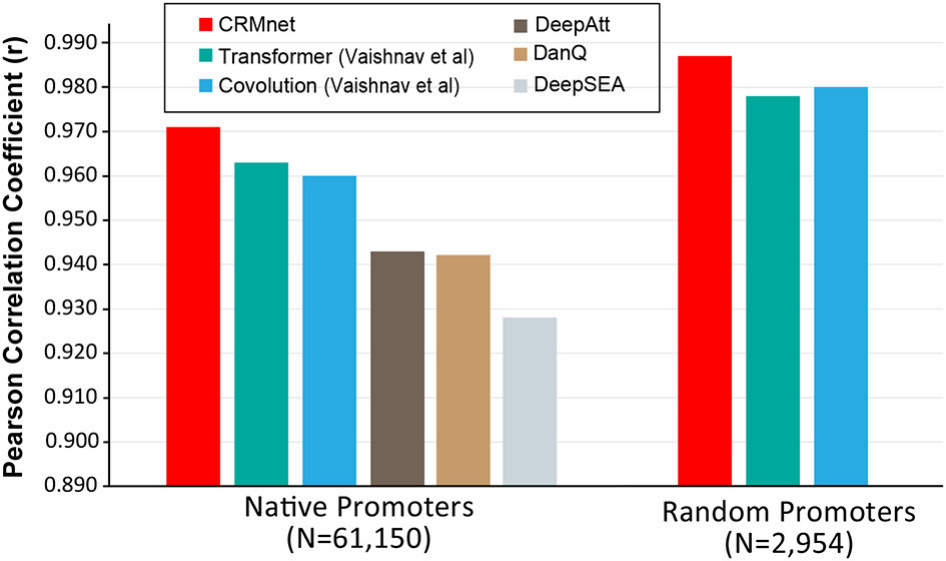
Benchmarking the CRMnet's performance against existing neural network architectures. The prediction performance of CRMnet on yeast native promoters and random promoters was compared with the transformer and CNN models from Vaishnav et al. (2022) and other existing DNNs [DeepAtt (Li et al., 2021), DanQ (Quang and Xie, 2016), and DeepSEA (Zhou and Troyanskaya, 2015)]. The performance of DeepAtt, DanQ, and DeepSEA on random promoters was not published in Vaishnav et al. (2022).

We next compared our model's performance with the benchmark models on the same test datasets. Our CRMnet model outperforms the benchmark transformer model proposed by Vaishnav et al. (2022) in both native and random promoter test datasets in both mediums (Figure 3), and also outperforms other existing deep learning models referred to in Vaishnav et al. (2022). The models DeepSEA (Zhou and Troyanskaya, 2015), DanQ (Quang and Xie, 2016), and DeepATT (Li et al., 2021) were utilized for performance comparison in the study by Vaishnav et al. (2022). As these three models were originally designed to solve the classification problem of predicting transcription factor binding sites, modifications were made to the input and output layers to adapt them to the regression problem of predicting gene expression from sequences, while maintaining the core architecture intact (Vaishnav et al., 2022). Specifically, our model achieved a Pearson correlation coefficient of 0.971 on native sequences, which is higher than the coefficient of 0.963 achieved by Vaishnav et al.'s transformer model and the coefficient of 0.960 achieved by Vaishnav et al.'s convolutional model. Additionally, our model achieved a Pearson correlation coefficient of 0.987 on random promoter sequences, which is again higher than the coefficient of 0.979 achieved by Vaishnav et al.'s transformer model and the coefficient of 0.980 achieved by Vaishnav et al.'s convolutional model. A detailed performance summary can be found in Supplementary Tables 2, 3.

### 3.2. Ablation study

**Figure 4:**
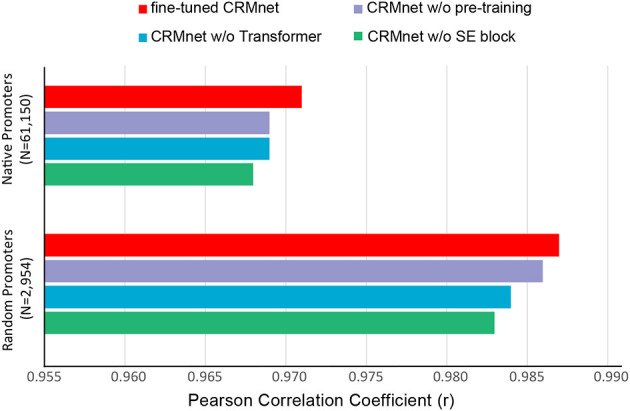
Prediction performance for ablation study. Comparisons of prediction performance of models tested on native promoters and random promoters are shown: the full model with transfer learning (fine-tuned CRMnet, red), the model without transfer learning (CRMnet without pre-training, purple), the model without transformer block (CRMnet without transformer, blue), and the model without squeeze excitation (SE) block (CRMnet without SE block, green).

To determine the contribution to the performance of the various components of our model, we performed an ablation study. Specifically, for each ablation experiment, we constructed a new model with the ablated block removed from the original CRMnet architecture and trained the new model using the same training dataset as the original CRMnet. We then evaluated the performance of our models using the native and random test datasets (complex medium only) (Figure 4).

Pre-training followed by fine-tuning on the complex medium training dataset (Figure 4, red bar) demonstrates substantial improvement compared with a model directly trained on the complex medium training set (Figure 4, purple bar), due to the larger training data set size and improved starting point for fine-tuning model training by transfer learning. Overall performance decreased when the transformer (Figure 4, blue bar) and squeeze excitation blocks (Figure 4, green bar) were removed from the model without transfer learning, particularly for the random dataset, indicating that both blocks contribute to the model's predictive performance.

### 3.3. Model interpretation

To explore the biological insights from our trained model, we used saliency maps to interpret the model by visualizing predictive motifs. Saliency maps based on gradient backpropagation have been commonly applied to highlight model-derived features in input data (Adebayo et al., 2018), and have been used to interpret the relationship between the input and prediction of the trained model, where a segment of the input with a higher saliency value indicates an influential region for the model's prediction (Eraslan et al., 2019). By combining the gradient values with the input sequences, also known as input-masked gradients, we can visualize the segments that significantly impact the model's prediction (Eraslan et al., 2019).

For comparison, we first searched for significant TF motifs using probabilistic motif discovery based on expression levels (see Section 4). We discovered the known yeast motifs associated with higher expression levels: NHP10 (High-mobility group (HMG) domain factors), REB1 (Myb/SANT domain factors), ABF1 (Basic helix-loop-helix factors (bHLH), AZF1 (C2H2 zinc finger factors), and RAP1 (Myb/SANT domain factors) were the top 5 motifs.

**Figure 5:**
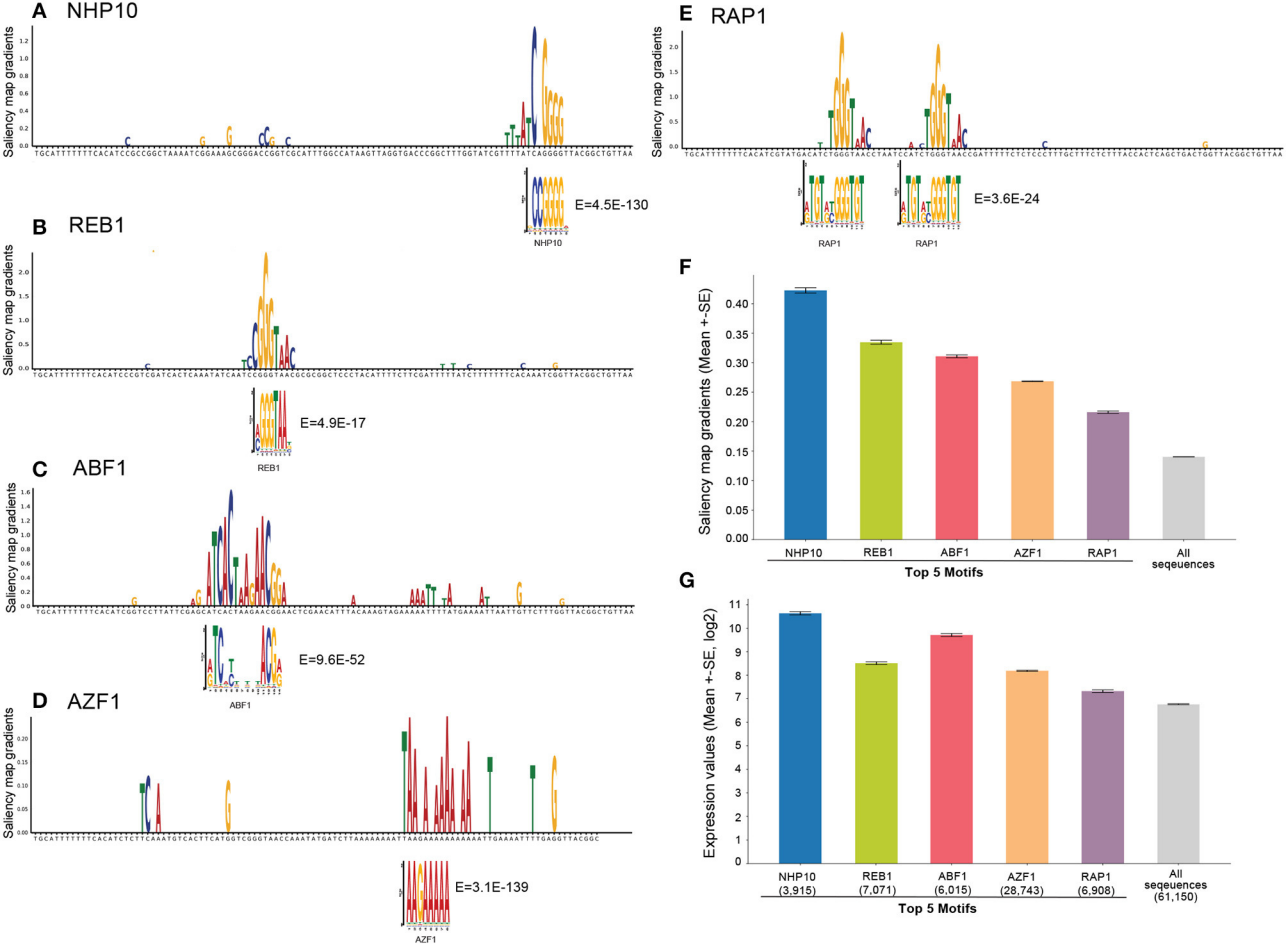
Model interpretation by saliency maps. **(A–E)** The top 5 yeast TFBS motifs detected by motif discovery: NHP10, REB1, ABF1, AZF1, and RAP1. Shown is an example sequence with its saliency map gradients over 80-nt for each motif, aligned with the known TF motif logo and *E*-values. **(F)** Mean saliency map gradients over these top 5 motif matches in yeast native sequences, and mean saliency map gradients over all sequences as the controls. **(G)** Mean expression levels of yeast native sequences containing these top 5 TF motifs, and all native sequences as the control.

We then visualized the input-masked gradients by plotting the saliency map logos generated from our fine-tuned model over yeast native sequences compared to these significant motifs from probabilistic motif discovery. To enhance the visualization, we applied a filter to eliminate gradients that were lower than the average gradient for each sequence and removed gradients with a low weight (less than 0.1). We also set the gradient values for adapter sequences to zero to eliminate the effect of the adapter sequences, which are the same across all input sequences, and to focus on potential motifs in the middle 80 nucleotide region. The results show that the saliency map matches the known yeast motif logos (Figures 5A–E and Supplementary Figure 2). To quantify this, we further calculated mean saliency map gradients over the positions in the sequences matched by these top 5 motifs and showed that these motifs are associated with substantially higher saliency gradients than the mean over all sequences as a control (Figure 5F).

Furthermore, we calculated the mean expression levels of yeast native sequences containing these top 5 TF motifs compared to all sequences as a control. The result shows that these motifs are associated with higher expression levels as expected (Figure 5G). Notably, the saliency gradients showed that the TF motifs associated with the highest expression levels contribute the most to the prediction of gene expression, supporting that our model extracts biologically meaningful features.

### 3.4. Training time comparisons

We next compared the training time between eight TPU V3 cores and eight GPU A100s under different batch sizes and precision settings (shown in Table 1). Training on the V100 GPU with batch size equal to 1,024 and default precision setting was used as the benchmark. The time-per-step is the average processing time to process one batch of data. The average epoch time represents how long it takes to run over all the data. The training time was estimated for the model to run 20 epochs without considering model convergence and the initialization time. For instance, if the local batch size is set to 4,096 and distributed training is conducted using 8 GPUs, the global batch size would be 4,096 × 8 = 32,768. In this scenario, the total required graphics memory without the mixed precision policy is 270.14 GB. However, if the mixed precision policy is implemented, the total required graphics memory is reduced to 142.17 GB. The blank value indicates the batch size is too big and over the accelerating hardware's memory limit.

**Table 1:** Summary of training speed for different batch sizes and accelerator hardware configurations.

**Local batch size**	**Hardware**	**Number of processor**	**Mixed precision**	**Time per step (s)**	**Average epoch (min)**	**Training time (h)**
1,024	V100 32G	1	float32	0.434	210.37	70.12
1,024	A100 80G	8	float32	0.187	11.81	3.94
	A100 80G	8	mixed float16	0.187	11.81	3.94
	TPU-V3	8	float32	0.172	10.86	3.62
	TPU-V3	8	mixed bfloat16	0.165	10.42	3.47
4,096	A100 80G	8	float32	0.675	10.65	3.55
	A100 80G	8	mixed float16	0.477	7.51	2.51
	TPU-V3	8	float32	0.726	11.46	3.82
	TPU-V3	8	mixed bfloat16	0.611	9.64	3.21
8,192	A100 80G	8	float32	1.337	10.54	3.51
	A100 80G	8	mixed float16	0.889	7.01	2.34
	TPU-V3	8	float32	-	-	-
	TPU-V3	8	mixed bfloat16	1.353	10.67	3.56
16,384	A100 80G	8	float32	-	-	-
	A100 80G	8	mixed_float16	1.718	6.76	2.25
	TPU-V3	8	float32	-	-	-
	TPU-V3	8	mixed bfloat16	-	-	-

The benchmark training time was calculated by training the model on complex medium data with a single V100 GPU, with a local batch size of 1,024 and no mixed precision policy. The final training time is based on training the model for 20 epochs without considering the model's convergence. The training time was left empty if the local batch was too large and exceeded the hardware's graphic memory limit. For distributed training on multiple hardware, we compared the training time between TPU v3-8 and DGX box with eight A100s, where each configuration contains eight accelerator hardware.

The study showed that using distributed training on multiple hardware accelerators can greatly reduce training time to manageable levels, cutting it down from 70 h to just 4 h. Additionally, using mixed precision in the training process can enhance GPU performance, particularly when utilizing large batch sizes, and can also decrease the amount of memory required. Furthermore, the study found that the most recent GPU, the A100, with 80 GB of graphics memory can handle larger batch sizes than TPU v3-8, which has 32 GB of memory per core.

## 4. Materials and methods

### 4.1. Data collection

In the study of Vaishnav et al. (2022), the yeast cells were grown under different mediums to exercise different metabolic pathways. Here, we used data from cells grown in the complex (yeast extract, peptone, and dextrose) and defined (lacking uracil) medium as specified in Vaishnav et al. (2022). The training data was downloaded from https://zenodo.org/record/4436477, containing 30,722,376 and 20,616,659 random sequences from complex and defined medium, respectively, with their expression values evaluated by Gigantic Parallel Reporter Assay experiment (Supplementary Table 1).

For model testing data, we used independent test sets drawn from experimental replicate datasets, which were generated in Vaishnav et al. (2022), consisting of native and random promoter sequences (*N* = 61,150 and *N* = 2,954, respectively) from complex medium and (*N* = 3,782 and *N* = 5,284, respectively) from defined medium (Supplementary Table 1). The test data was downloaded from GitHub repository at https://github.com/1edv/evolution.

### 4.2. Model training setup

We first trained individual models using data from complex medium and defined medium separately. Specifically, 30,722,376 random sequences from the complex medium and 20,616,659 random sequences from the defined medium were used to train the individual models. For the pre-trained model, we evenly sampled data from both media. In total, 51,339,035 sequences and their experimentally measured expression levels were used for pre-training the model. We then further trained the pre-trained model on complex and defined medium separately in the fine-tuning process.

The model's performance was assessed using independent test datasets as described above, and none of the sequences in the test datasets were used during model training. It is important to note that the test data library was measured in separate experiments from the training data and that the test data library contains fewer sequences than the experiments used to generate the training data. As a result, the expression value associated with each sequence was precisely measured in the test data (by averaging 100 yeast cells per sequence).

### 4.3. Data pre-processing

The initial input sequences have a length of approximately 110 nucleotides. To standardize the length of the sequences, we employed the TensorFlow 2 function “tf.keras.preprocessing.pad_sequence” to adjust the sequences to a fixed length of 112 nucleotides. This length was chosen as it has multiple factors of two, which makes it more convenient for implementation. It allows for easy reduction or expansion of the input size by half or two times at different levels in the model. Specifically, sequences longer than 112 nucleotides were truncated from the end, while sequences shorter than 112 nucleotides were padded with the nucleotide “N” at the end. Truncating longer sequences may result in information loss, however, in our case, we have millions of sequences with over 96% of them having a similar length of 110 nucleotides, with a deviation of 2 nucleotides. The detailed distribution of the input sequence lengths can be found in Supplementary Figure 3. This indicates that the risk of information loss is minimal in our case. Pre-processing the sequences to have the same length is necessary for the input to the convolutional neural networks. We then used one-hot encoding to encode the nucleotides based on the order of “A”, “C”, “G”, and “T”. Specifically, we used *tf.keras.layer.StringLookup* function to encode the input sequences and define the vocabulary as [“A”, “C”, “G”, “T”] while characters not in the vocabulary (i.e., “N”) are encoded in the fifth dimension. Therefore, “A”, “C”, “G”, “T”, and “N” are encoded to [1,0,0,0,0], [0,1,0,0,0], [0,0,1,0,0], [0,0,0,1,0], and [0,0,0,0,1], respectively.

### 4.4. Model execution

Since we mainly used TPU v3-8 VM to train our model, which contains eight tensor processing cores, we set the global batch size to 8192, where each TPU core is assigned 1024 samples. To accelerate the efficiency of feeding data into the model, we prefetch the training data into the memory using the *tf.data.Dataset.prefetch()* function. This operation reduced the latency and improved the data pipeline throughput. We set the buffer size for prefetching the data equal to *tf.data.AUTOTUNE*, which will optimize the number of data prefetched automatically.

We applied the training process of our model to two different hardware: TPUs and GPUs. For Tensor Processing Units (TPU), provided by Google TPU Research Cloud, we trained the model with the TPU v3-8 virtual machine. The TPU v3-8 virtual machine comes with 8 processing cores. So, we set the global batch size equal to 8,192, in which each core is allocated a local minibatch size equal to 1,024. For the GPUs, we trained the model using eight Nvidia DGX A100 GPUs provided by Australian National Computational Infrastructure. Thus, following the same setup as TPUs, we set the global batch size equal to 8,192 to ensure each A100 GPU processed a minibatch with 1,024 samples in parallel.

We used Huber loss to calculate the difference between predictions and true values for the loss function. The formulation of Huber loss can be expressed as follows:


(1) $$\begin{array}{l} L_{\delta }=\{\begin{array}{cc} \frac{1}{2}{\left(y-ŷ\right)}^{2} & if|\left(y-ŷ\right)|<\delta \\ \delta \left(|y-ŷ|-\frac{1}{2}\delta \right) & otherwise \end{array} \end{array}$$

The Huber loss function is a combination of the mean absolute error (MAE) and mean squared error (MSE) loss functions, with a control factor, delta (by default, delta is set to 1). When the difference between the predicted label and the true label is less than delta, the Huber loss function behaves like the MSE, which is more sensitive to smaller loss values due to the quadratic function. However, when the difference between the predicted label and the true label is greater than delta, the Huber loss function uses the MAE, which is less sensitive to large loss values as it reduces the impact of outliers (Huber, 1992). We use Adam optimizer to optimize the Huber loss function. For the learning rate scheduler, we set a learning rate warm-up in the first 10 epochs, which gradually increase the learning rate of the optimizer from 0.0001 × NUM of HARDWARE (i.e., 0.0008) to 0.001 × NUM of HARDWARE (i.e., 0.008). A cosine decay learning rate scheduler was then used to gradually reduce the learning rate to 0.0001 × NUM of HARDWARE (i.e., 0.0008). To avoid overfitting, an early stop call-back function was used. This call-back function monitors the model's performance over the validation dataset. If the model's validation R-square value was not improved in the most recent ten epochs, it stopped training and restored the model weight with the best performance over validation data.

We used the Tensorflow 2's *tf.distribute.MirroredStrategy* for distributed training on a DGX A100 box, which contains eight A100 GPUs. Additionally, we used the *tf.distribute.TPUStrategy* for training on a TPU v3-8 virtual machine, which has eight tensor cores. Both strategies are synchronous training processes intended to distribute training across multiple processing units on a single machine. The synchronous strategy first copies all of the model's variables to each processor. During training, each processor is assigned a portion of the training data, which is used to update the trainable parameters on that processor. This distributed training method, a form of data parallelism, allows each device to handle a different part of the data. The gradients from each processor were then fused using all-reduce and The resulting values are synchronized to all instances stored in each processor. Since our model training does not require high precision and training on TPUs automatically uses float16, we used the mix precision policy by setting up precision equal to mixed_float16 for training on GPUs. This policy improves our model training speed on GPUs without losing accuracy.

We further compared the training speed between A100 GPUs and TPU V3-8 with different local batch sizes and mixed precision policy. To reduce the impact of other factors, such as the time used to build the computational graph, we exclude the time reported in the first epoch and take the maximum value from the time-per-step column. The time-per-step report is the average time the hardware processes each batch in one epoch. The training speed comparison is shown in Table 1. Note that the final training time is an optimistic estimation for training the model for 20 epochs, which doesn't guarantee the model convergence and doesn't consider the overhead time used for inter-core communication.

For the software and packages, we used Python 3.8.10 to write the code for training and evaluation. For data pre-processing, we used NumPy 1.22.1 and Pandas 1.5.0 packages. We used TensorFlow 2.8.0 framework to implement and train the neural network. Functions from TensorFlow Addons 0.16.1 and SciPy 1.9.3 were used to evaluate model performance. Matplotlib 3.6.1 and Seaborn 0.12.1 were used for visualization.

### 4.5. Motif discovery

We used the probabilistic motif discovery tool MEME (Bailey et al., 2015) in “differential enrichment mode” to detect the motif enrichment in the top 2,000 sequences with high gene expression against the bottom 2,000 sequences with low gene expression. We used FIMO in the MEME suite to search for motif hits in yeast native promoter sequences.

We further used two ranking-based methods, Discovering Ranked Imbalanced Motifs using Suffix Trees (DRIMust) (Leibovich et al., 2013) and rGADEMm (Mercier et al., 2011), to determine the *de novo* motifs in a ranked list of sequences, which were ranked from high to low expression values. DRIMust uses suffix trees to identify overrepresented motifs in the top-ranked sequences and further evaluates the obtained k-mers by minimum-hypergeometric (mHG) approach (Leibovich et al., 2013). rGADEM combines spaced dyads and an expectation-maximization (EM) algorithm. The spaced dyads are identified by their overrepresentation in the input sequences, and a genetic algorithm is further employed to mark them significant and to declare them as motifs (Mercier et al., 2011). We used Bioconductor packages “TFBStools” (Tan and Lenhard, 2016), “JASPAR” (Castro-Mondragon et al., 2022), to match the identified motifs with known JASPAR Yeast motifs.

We selected the top 5 motifs ranked by *E*-value in MEME that were reproduced by the rank-based methods.

## 5. Conclusion

In this study, we introduced CRMnet, a novel neural network architecture that accurately predicts the gene expression levels driven by yeast promoter sequences. The architecture of the model is inspired by the prior biological knowledge that promoter sequences contain multiple contiguous TFBS motifs which together coordinately regulate gene expression. Our model is an improvement over (Vaishnav et al., 2022) transformer model because it utilizes a U-Net inspired architecture to capture regulatory sequences in the encoder. This entails using initial convolutional neural network layers as a feature extractor to identify the contiguous regions denoting individual TFBS motifs, followed by transformer encoders to weight the influential motifs and, importantly, detect correlative patterns between motifs predicting gene expression. The decoder stage propagates the feature maps at base-level precision, potentially improving model interpretation precision. Additionally, our model uses transfer learning, i.e., it was pre-trained on a large dataset and then fine-tuned, and a multi-layer perceptron and skip connections in the decoder for improved prediction of gene expression.

Our ablation studies of the CRMnet model demonstrated the potential for improvements in predictive performance for a given biological problem by the design of custom DNN architectures. In particular, augmentation of a model with a combination of CNN and additional transformer stages guided by training and testing results on large high-throughput datasets can give useful increments in performance.

Importantly, high performance DNN models extracting dependency information *via* attention mechanisms allow for biological insights through model interpretation. In this study, we visualized regions of key importance for transcriptional expression regulation by plotting the saliency map over the input yeast DNA sequences. Notably, we found that the logo plots constructed from saliency maps over the input sequences are correlated with the sequence motifs of known yeast transcription factors. The combination of future improvements in DNN model architectures and model interpretation methods, in concert with appropriately designed high-throughput synthetic experimental data, will be key enablers for future biological discoveries and elucidation of subtle regulatory signals, from enhancer regions to post-transcriptional regulatory signals.

## Data availability statement

The code is available on GitHub at https://github.com/jiayuwen/CRMnet. The final model and prepossessed data are available at https://zenodo.org/record/7375243#.Y4gDjS0RoUE.

## Author contributions

KD conducted all machine learning coding and drafted the manuscript. GD collected the data and performed the motif analysis. BP and JW supervised the project, provided the guidance, discussed the results, and revised the manuscript. All authors read the current manuscript and approved the submitted version.
