# Supplementary material for: CRMnet: A deep learning model for predicting gene expression from large regulatory sequence datasets
Source: Front Big Data. 2023 Mar 14;6:1113402. doi: 10.3389/fdata.2023.1113402 (PMC10043243; doi:10.3389/fdata.2023.1113402)
Supplement: Supplementary file 1 [file Data_Sheet_1.PDF]

Supplementary Material

SUPPLEMENTARY TABLES AND FIGURES

Tables

Table S1. Summary of yeast training/testing datasets.

Data type	Usage	Yeast medium	#sequences
Random Promoter Sequences	training/testing	Complex (YPD)	30,722,376
Random Promoter Sequences	training/testing	Defined (SD-Uracil)	20,616,659
Native Yeast Promoter Sequences	testing	Complex (YPD)	61,150
Random Promoter Sequences	testing	Complex (YPD)	2,954
Native Yeast Promoter Sequence	testing	Defined (SD-Uracil)	3,782
Random Promoter Sequences	testing	Defined (SD-Uracil)	5,289

Table S2. Summary of benchmark models performance on test native sequences (N = 61,1150).

Model	r	MSE	RMSE	MAE
DanQ	0.942	3.362	1.834	**1.488**
DeepAtt	0.943	3.687	1.920	1.574
DeepSEA	0.928	3.804	1.950	1.579
Convolution (Vaishnav et al)	0.960	3.270	1.808	1.490
Transformer (Vaishnav et al)	0.963	3.965	1.991	1.689
CRMnet	**0.971**	**3.200**	**1.789**	1.509

r: Pearson correlation coefficient; MSE: mean squared error; RMSE: root mean squared error; MAE: mean absolute error.

Table S3. Summary of benchmark models performance on test random sequences (N = 2,954).

Model	r	MSE	RMSE	MAE
DanQ	0.963	1.642	1.281	1.013
DeepAtt	0.958	1.721	1.312	1.005
DeepSEA	0.953	2.194	1.481	1.224
Convolution (Vaishnav et al)	0.980	1.167	1.080	0.857
Transformer (Vaishnav et al)	0.978	1.425	1.194	0.894
CRMnet	**0.987**	**1.012**	**1.006**	**0.770**

r: Pearson correlation coefficient; MSE: mean squared error; RMSE: root mean squared error; MAE: mean absolute error.

Figures

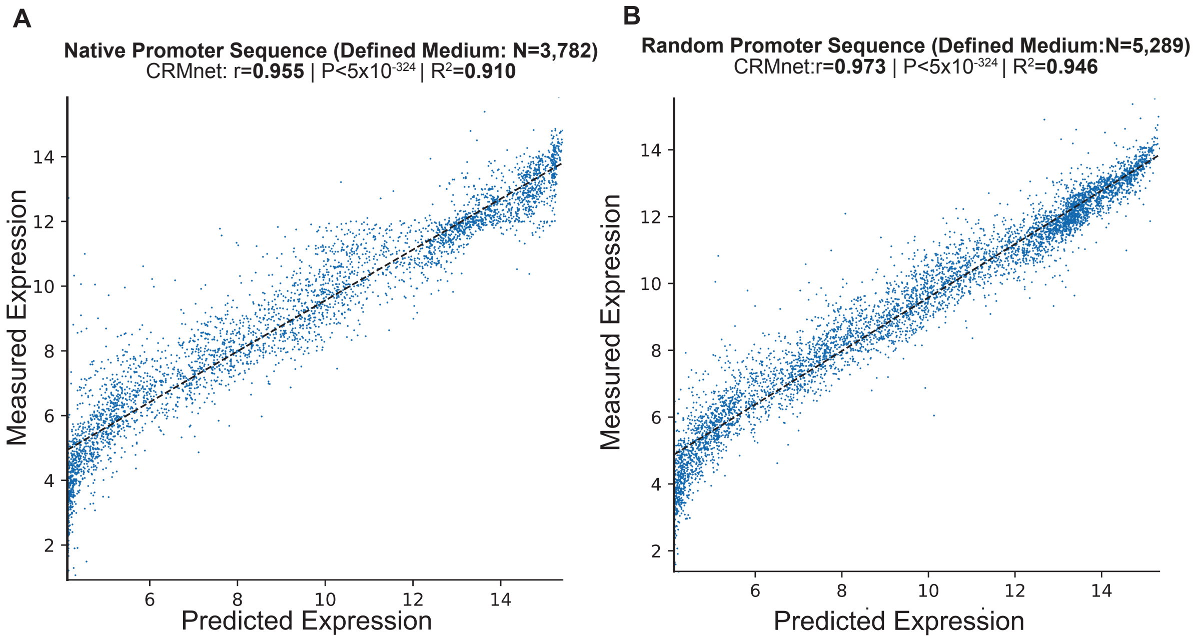

Figure S1. Prediction of expression from yeast native sequences in defined medium from the fine-tune CRMnet. Fine-tuned CRMnet tested on A: native promoter sequences; and B. random promoter sequences. The y-axes represent measured expression levels, while the x-axes represent predicted expression levels. As a benchmark, the model performance metrics of the Pearson r value, associated two-tailed p-values, and R-square for the transformer model from Vaishnav et al. (2022)
showed: A: r=0.950, P $< 5 \times 10^{-324}$, R^2=0.900; B: r=0.968, P $< 5 \times 10^{-324}$, R^2=0.937

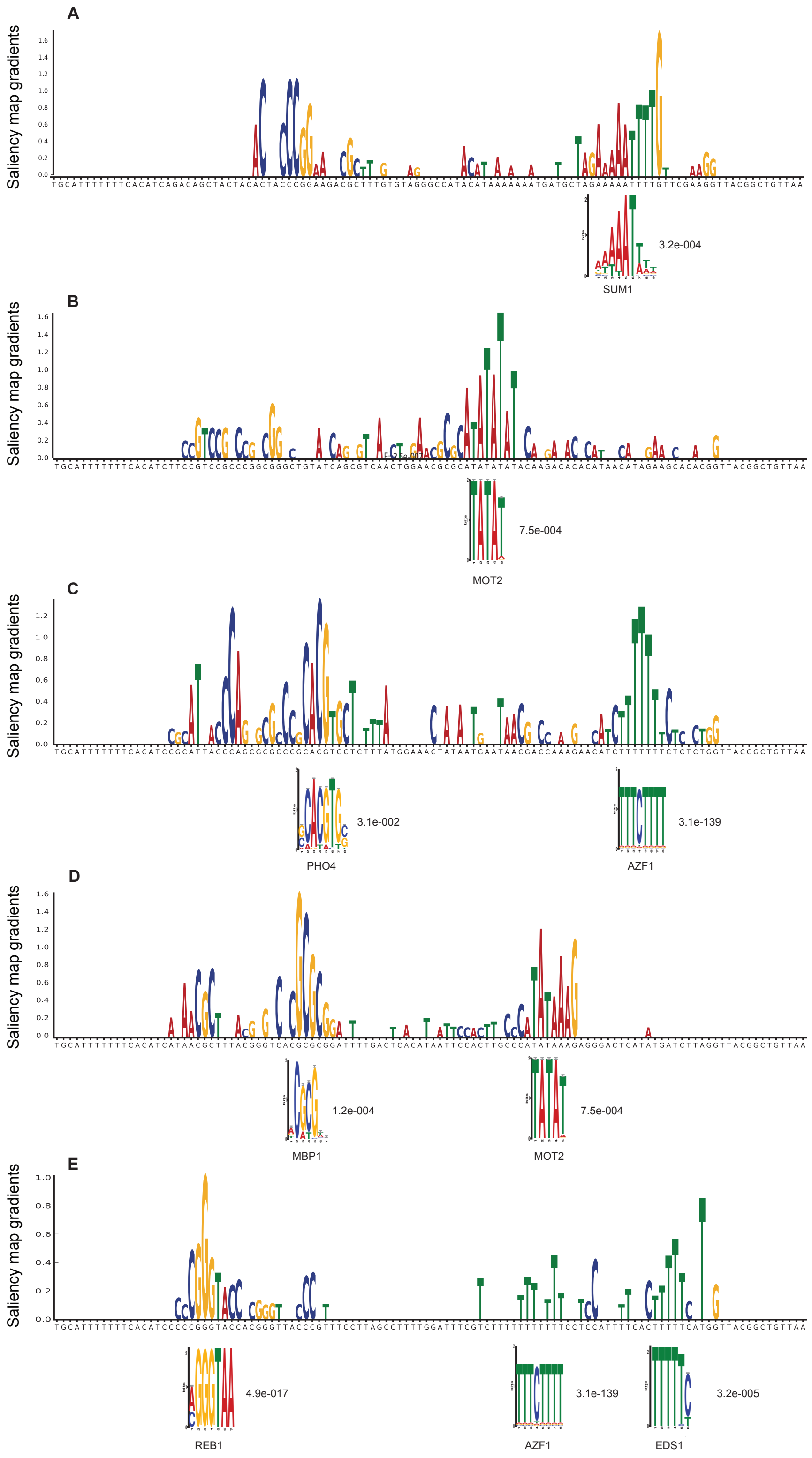

Figure S2. Model interpretation by saliency maps. Additional TF motifs detected by motif discovery (E-values < 1E-3). Shown is an example sequence with its saliency map gradients over 80-nt for each motif, aligned with the known TF motif logo and E-values.

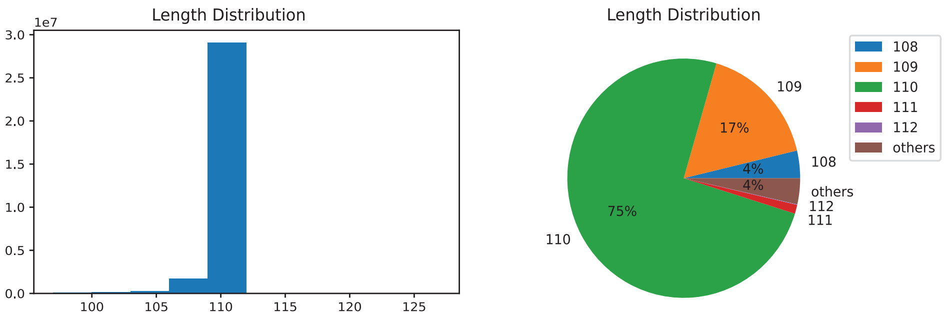

Figure S3. Input sequences length distribution. Length distribution of the input sequences where over 96% of the input sequences have the input length of $110nt \pm 2nt$.

REFERENCES

Vaishnav, E. D., de Boer, C. G., Molinet, J., Yassour, M., Fan, L., Adiconis, X., et al. (2022). The evolution, evolvability and engineering of gene regulatory dna. *Nature* 603, 455–463
